# Exploring the skin as an open window onto neurodegenerative diseases

**DOI:** 10.1186/s40035-025-00522-4

**Published:** 2025-11-27

**Authors:** Francesca Lozzi, Emanuela Camera, Giorgia Cardinali, Anna Di Nardo

**Affiliations:** Laboratory of Cutaneous Physiopathology and Integrated Center of Metabolomic Research, San Gallicano Dermatological Institute, IRCCS, 00144 Rome, Italy

**Keywords:** Neurodegenerative diseases, Biomarkers, Early diagnosis, Skin, Non-invasive techniques

## Abstract

Neurodegenerative diseases (NDDs), including Parkinson's disease and Alzheimer's disease, are major age-related disorders characterized by progressive neuronal degeneration and a decline in cognitive and motor functions. Managing NDDs poses an increasing healthcare challenge as the global population ages. The onset of NDDs is linked to protein misfolding, oxidative stress, dysfunction of mitochondria and lysosomes, and neuroinflammation. Clinical manifestations of NDDs only appear after substantial neuronal damage has already occurred. This underscores the urgent need for accessible tissue biomarkers to enable early diagnosis, disease monitoring and assessment of therapeutic efficacy. The skin has emerged as a valuable peripheral indicator of neurodegeneration, sharing embryological origin, gene expression profiles, protein alterations and cellular dysfunctions with the brain. Notably, pathological protein deposits, which are hallmarks of NDDs, such as beta-amyloid, tau proteins, and oligomeric alpha-synuclein, have been observed in the skin. Increasing evidence links NDDs with various pathological skin conditions, including melanoma and inflammatory diseases. This review aims to explore the potential of the skin as a window into neurodegenerative processes at an early stage, before clinical signs arise. The main advantages of using skin as a source of NDD biomarkers are its accessibility and the minimally invasive sampling methods such as stratum corneum collection, sebum and volatile compounds analysis, and biopsies. Immunohistochemistry and omics approaches applied to skin samples provide valuable insights into NDD pathophysiology and facilitate biomarker discovery for early diagnosis and disease monitoring. NDDs are multisystemic disorders and new findings in skin research highlight the value of peripheral tissues for investigating central nervous system alterations enabling earlier neuroprotective interventions.

## Background

Neurodegenerative diseases (NDDs) are a heterogeneous group of neurological disorders characterised by progressive degeneration of neurons, primarily in the central nervous system (CNS) [1], but also in the peripheral nervous system (PNS) [2, 3]. The most common and well-characterised NDDs are Alzheimer's disease (AD), Parkinson's disease (PD), amyotrophic lateral sclerosis (ALS), and Huntington's disease (HD), which primarily affect the aging population [1, 4, 5]. Although the exact aetiology of NDDs remains incompletely elucidated, current evidence indicates that neuronal dysfunction and loss result from a combination of genetic, environmental, and lifestyle factors [6, 7]. While each NDD possesses specific hallmarks [8], the majority exhibit common pathogenic events that underlie their onset and progression. A shared key feature of NDDs is the accumulation of misfolded proteins, which leads to neuroinflammation, impaired synaptic signaling, and neuronal cell death [9–12]. Impairment of protein homeostasis mechanisms, including autophagy and the ubiquitin–proteasome system, is associated with the accumulation of misfolded proteins in the brain [8, 13, 14]. Other features of NDDs include oxidative stress, mitochondrial dysfunction, neuroinflammation, and genetic and epigenetic modifications.

As the largest organ in the human body, the skin shows changes due to the ageing process and can therefore mirror age-related pathological conditions, specifically neurodegenerative processes. The skin is closely connected to the CNS. In fact, they have the same embryonic origin: during gastrulation, the dorsal ectoderm differentiates into the neural crest, while the ventral ectoderm differentiates into the epidermis. This establishes and maintains bidirectional communication through the so-called skin-brain axis [15, 16]. This connection is also elicited by signals from the endocrine and immune systems, as well as microbial factors. These elements contribute to regulating fundamental skin processes, such as the stress response, inflammation, barrier function in both skin and CNS, and sensation [15]. The skin innervation is an integral part of the PNS, enabling a two-way exchange of signals. In particular, skin autonomic nervous system (ANS) fibres (mostly sympathetic) control the dilation or constriction of blood vessels, sweating, defence mechanisms against inflammation or infection, and wound healing. On the other hand, sensory fibres enable the CNS to receive input in response to sensory stimuli such as touch, temperature, and pain [17, 18]. Because of the dynamic interaction between the skin and the brain, these two organs influence each other [19].

In this review, we summarize the clinical features, aetiological mechanisms, and biomarkers of the most common NDDs, focusing specifically on correlations with pathological skin conditions. We aimed to highlight valuable cutaneous research findings that explore the potential of the skin as a model for studying age-related neurodegeneration, as well as a source of predictive biomarkers.

## The brain-skin axis: pathogenetic mechanisms of neurodegenerative diseases and related dermatological aspects

The gradual development of NDD symptoms, combined with delayed appearance of many clinical signs until significant neuronal damage occurs, makes early diagnosis challenging and limits opportunities for timely intervention and care for patients. Therefore, identifying specific biomarkers is essential for making pre-symptomatic diagnoses, predicting disease progression, tracking development, and monitoring patient response to pharmacological therapies [20, 21]. Beyond enabling early-stage disease identification, an effective biomarker should exhibit exceptional accuracy and sensitivity. Biomarkers for NDDs are detected in various biofluids, including blood, cerebrospinal fluid (CSF), saliva, urine, and peripheral tissues such as the salivary glands, gut mucosa, and skin [21–23].

In recent years, growing evidence has highlighted the complex bidirectional communication between the CNS and the skin. Identifying biomarkers of NDDs in skin samples represents a promising non-invasive approach for early detection and monitoring of neurodegenerative processes. Various studies have revealed the presence of NDD-related misfolded proteins in skin biopsies from different body areas in patients with NDDs [19, 24–27]. Furthermore, in vitro research has demonstrated shared pathogenic mechanisms between cutaneous and neuronal cells [28–31]. Additionally, pathological brain conditions have been linked to certain skin disorders such as psoriasis, seborrheic dermatitis, bullous pemphigoid, rosacea, and melanoma, suggesting potential systemic interactions [32–34] (see Table 1).

**Table 1:** Skin comorbidities associated with major neurodegenerative diseases

NDDs	Skin comorbidities	References
Alzheimer’s disease	Psoriasis	[61, 62, 67]
	Melanoma	[54, 55, 58, 59]
Parkinson’s disease	Melanoma	[123–130, 135, 151]
	Bullous pemphigoid	[131, 132, 135, 141, 143–145, 151]
	Seborrheic dermatitis	[76, 131, 132, 135, 146, 151–156]
	Rosacea	[131, 132, 135, 137, 151]

Therefore, skin manifestations are increasingly recognised as potential early indicators of comorbidities associated with NDDs. Improving diagnosis and monitoring, and identifying new therapeutic targets, require a better understanding of the dermatological aspects connected with neurodegenerative processes.

## AD

AD is one of the most common types of dementia in older adults, mainly affecting memory, understanding, thinking, and behaviour [32–34]. The hallmarks of AD are the build-up of plaques containing β-amyloid (Aβ) proteins and twisted filaments of tau as well as the presence of hyperphosphorylated isoform Aβ_42_ and phosphorylated tau proteins (p-tau) [35–39]. Familial AD is mainly associated with mutations in *PSEN1*, *PSEN2*, and *APP* genes which lead to an excessive production of Aβ, or total tau (t-tau) and p-tau proteins [40, 41]. Aβ positron emission tomography is commonly used in clinical practice to quantify and localise the accumulation of Aβ in the brains of patients with AD. CSF biomarkers can be used to achieve a reliable diagnosis, similar to PET. Analysing the Aβ_42_/Aβ_40_ ratio in plasma is another method of detecting amyloid plaques [42, 43]. Three phosphorylated forms of tau proteins involved in AD have recently been identified in plasma: p181-tau, p217-tau, and p231-tau [44]. Current studies have clearly demonstrated that the p217-tau detected in blood is an excellent indicator of disease status, exhibiting high specificity and sensitivity to disease-related clinical changes [45], and enabling discrimination between AD and other NDDs [46].

Various studies have investigated the presence and deposition of Aβ and tau proteins in human skin. Since the 1990s, the presence of Aβ has been demonstrated in skin biopsies from patients with Down syndrome and AD in the dermis, epidermis, and blood vessels [47]. More recently, deposits of Aβ_42_, p-tau, and t-tau have been confirmed in human keratinocytes using double immunostaining. However, the differences between AD patients and healthy controls were not statistically significant [27]. AD patient-derived fibroblasts secrete more Aβ than those derived from healthy controls [48]. Skin fibroblasts from control patients develop an AD phenotype after Aβ treatment [49]. Furthermore, Aβ accumulated in the fibroblasts of AD patients induces changes in cell morphology, and impairs skin elasticity [50, 51].

Some evidence suggests a connection between AD and melanoma. Melanoma is a malignant tumour that originates in melanocytes, cells responsible for producing melanin. Melanoma is the most aggressive skin cancer and, if not detected early, metastasizes rapidly [52, 53]. Melanoma cells secrete Aβ, which promotes their proliferation and survival [54], and accumulates into extracellular amyloid aggregates reminiscent of those in AD [55]. Sporadic, late-onset AD [56, 57] is strongly associated with the *APOE* ε4 allele [58], which paradoxically has been shown to exert anti-tumor effects in melanoma, thereby enhancing survival [59]. Nevertheless, the relationship between melanoma and AD remains unresolved, reflecting the inconclusive nature of current evidence.

Neuroinflammation is a key feature of NDDs, characterized by activation of microglia and astrocytes, as well as the infiltration of mononuclear phagocytes and T lymphocytes. The subsequent release of proinflammatory cytokines (e.g., tumor necrosis factor-alpha [TNF-α], interleukin [IL]-1β, and IL-6), chemokines, and reactive oxygen species (ROS) increases blood–brain barrier (BBB) permeability and migration of peripheral immune cells into the CNS, priming the immune response. Chronic inflammation likely contributes to neuronal dysfunction and progressive neurodegeneration preceding AD. Aβ is proposed to function as a damage-associated molecular pattern activating toll-like receptors or NOD-like receptors. This interaction promotes the activation of nearby microglia and the release of inflammatory factors. Insufficient clearance of Aβ aggregates leads to persistent inflammation and sustained release of neurotoxic substances [10, 11]. A possible question in this context is how peripheral inflammation influences NDDs.

Several studies have examined the link between inflammatory skin conditions such as psoriasis, and the development of AD. Psoriasis is a chronic autoimmune skin disorder characterized by erythematous, scaly plaques typically affecting the scalp, palms, knees, elbows, and lower back [60]. Psoriasis biomarkers include keratins related to keratinocyte differentiation (K1 and K10) and activation (K6 and K16), as well as pro-inflammatory cytokines (IL-6, IL-8, IL-18, and TNF-α). A cohort study reported an increased risk of AD among patients with psoriasis who had not received systemic therapy [61]. Similarly, a large population-based analysis suggested that inflammatory disorders characterized by systemic TNF-α activity, including psoriasis, may elevate AD risk, whereas anti-TNF-α therapy appears to mitigate this effect [62]. In contrast, other studies have found no significant association between psoriasis and the risk of AD or dementia [63].

Because stress and inflammation impact both neurological function and skin health, investigating the hypothalamic–pituitary–adrenal (HPA) axis in neurodevelopmental disorders and its link with skin is relevant. Under stress, the hypothalamus releases corticotropin-releasing hormone, triggering the pituitary gland to produce adrenocorticotropic hormone, which then stimulates cortisol release from the adrenal glands. The HPA axis regulates the body's stress response via glucocorticoids like cortisol [64]. Dysfunction of the HPA axis is linked to NDDs, including AD. Patients with AD exhibit elevated cortisol levels, reflecting chronic stress and sustained activation of the HPA axis. Prolonged exposure to cortisol promotes the buildup of amyloid plaque [65].

Interestingly, the skin has a local HPA axis that closely mirrors the systemic one. Keratinocytes produce the same hormones and express corresponding receptors. This local axis helps the skin respond to stress, preserving cellular homeostasis and barrier function [66]. Although research on the role of the HPA axis in skin diseases is limited, dysfunctions have been associated with atopic dermatitis and psoriasis, and correlated with disease severity [67]. Persistent high levels of cortisol may indicate HPA axis dysfunction in both AD and skin conditions. Non-invasive methods, such as sensitive skin sensors that detect cortisol in sweat, offer a promising tool for monitoring these changes [68].

Altered metabolic pathways are linked to various diseases, including NDDs like AD [69, 70]. Lipidomic analysis of blood from AD patients suggests that certain lipids may play a role in disease development [71–73]. These metabolic changes in the brain may also appear in the skin, connecting them to dermatological symptoms [74]. Seborrhoeic keratosis, an age-related skin condition common in areas with many sebaceous glands, is associated with over-expression of *APP*, which is a key factor in the development of senile AD [75]. These findings emphasize the potential of lipidomic approaches in profiling lipid changes and discovering new lipid-based biomarkers for NDDs. To support this, lipidomic analysis has been performed on sebum, an easily accessible biological fluid that can be obtained through non-invasive methods. Briganti et al. analyzed the sebum of AD patients and found decreased lipid levels and less diversity of lipid species compared to healthy controls [76].

## PD

PD is the second most common neurodegenerative disorder after AD. It is characterized by motor symptoms, including tremor, rigidity, and bradykinesia, as well as non-motor manifestations such as sleep disturbances, gastrointestinal dysfunction, and dermatological changes [77–79]. Although most cases are sporadic, mutations in genes such as *SNCA*, *LRRK2*, *PINK1*, *PARK2*, *DJ-1*, and *GBA* are associated with familial and early-onset forms, disrupting mitochondrial function, mitophagy, protein degradation pathways, and oxidative stress responses, thereby promoting neuronal degeneration [80–83].

The neuropathological hallmark of PD is the accumulation of misfolded α-synuclein (α-Syn) into oligomers and fibrils that deposit within Lewy bodies in the brain [84, 85]. α-Syn aggregates are also found in peripheral tissues, including salivary glands, pancreas, the gastrointestinal tract, and skin [86], often preceding motor symptoms [87]. Early gastrointestinal symptoms like constipation and incontinence suggest a peripheral onset of PD [88], supporting the hypothesis that PD may originate in the gut and spread to the CNS via the vagus nerve and enteric nervous system [89]. Alterations in the gut microbiota further reinforce this view: patients with PD consistently show reduced anti-inflammatory species and increased pro-inflammatory taxa, leading to impaired production of short-chain fatty acids, increased intestinal permeability, and neuroinflammatory signaling [90–93]. These findings suggest that microbial shifts and their metabolites may serve as early biomarkers of PD, though a causal link to central pathology remains to be established.

Abnormal α-Syn deposits contribute to dopaminergic neuron degeneration and loss in the substantia nigra, and are observed in diverse biological fluids [82, 94]. However, findings on the levels of phosphorylated (p-α-Syn) and oligomeric (o-α-Syn) forms in the CSF and blood of PD patients are contradictory and inconclusive. Some studies have reported elevated levels of p-α-Syn and o-α-Syn in the CSF [23, 95], while blood levels show contradictory results, ranging from no change to increases of α-Syn in serum and plasma [96–100]. These contradictory findings underscore the need for exploring a reliable skin marker.

In recent years, the skin has emerged as a promising source of PD biomarkers, relevant to both diagnosis and disease pathogenesis. Early studies identified the presence of α-Syn in limited skin regions, such as the chest, in PD patients [101]. Further investigations expanded these findings, detecting pathological skin α-Syn in additional anatomical sites, including the scalp [102], abdomen [103], cervical skin [25, 103, 104], forearms [105] and legs [25]. Immunohistochemical analyses showed that α-Syn aggregates, particularly the phosphorylated form, are mainly localized at cutaneous nerve endings [106–108], but have also been found in epidermal cells, including keratinocytes and melanocytes [109, 110].

The effects of α-Syn have also been studied in vitro. Oliveira et al. demonstrated that o-α-Syn inhibits keratinocyte proliferation in the basal epidermal layer, promoting epidermal thinning. They also showed that o-α-Syn induces nuclear translocation of NF-κB in keratinocytes, resulting in increased production of pro-inflammatory cytokines such as IL-1β and TNF-α. This may lead to epidermal degeneration and impairment of skin mechanical properties [30]. Additionally, Kinnart et al. demonstrated that α-Syn overexpression in primary human fibroblasts disrupts mitophagic flux, impairing the clearance of damaged mitochondria [111]. Similar effects have been observed in neurons. These findings are supported by a study of primary fibroblasts from PD patients, which showed an accumulation of autophagosomes and autolysosomes, impaired mitochondrial activity and high ROS levels [112]. Overall, these results suggest that the skin reflects molecular alterations associated with synucleinopathies, opening up new possibilities for biomarker discovery through non-invasive methods.

Differentiating PD from atypical parkinsonisms (APs) is clinically challenging due to symptom overlap, especially in the early stages of the disease [113–115]. APs include progressive supranuclear palsy (PSP), multiple system atrophy (MSA), cortico-basal degeneration (CBD) and dementia with Lewy bodies [116]. APs typically progress faster and respond poorly to levodopa [115, 117, 118], and unlike PD, rarely feature prominent rest tremor or loss of smell [113].

Identifying α-Syn in skin offers a promising diagnostic tool. Unlike PD, PSP and CBD are distinguished by tau protein accumulation in neurons, rather than α-Syn, despite overlapping motor symptoms [115]. MSA, also a synucleinopathy, features α-Syn accumulation mainly in oligodendrocytes [119]. Some studies have demonstrated higher levels of α-Syn in skin biopsies of PD patients compared to those with AP, PSP or CBS, and the absence of α-Syn in healthy controls. The use of non-invasive techniques for differentiating PD from APs may improve diagnostic accurancy and reduce false-positiveness [110, 120].

Interestingly, a relationship has been hypothesized among α-Syn, melanin, and neuromelanin. α-Syn has been shown to bind directly to pigments during neuromelanin synthesis in dopaminergic neurons, preceding the depletion of neuromelanin [121]. Given the structural and functional similarities between melanin and neuromelanin, it is hypothesized that genes involved in skin pigmentation and melanogenesis may affect the vulnerability of dopaminergic neurons [122].

It has been reported that α-Syn and the PD-related oncogene DJ-1 are overexpressed in primary and metastatic cutaneous skin melanoma. α-Syn deposits have been found in metastatic melanoma lymph nodes, and its overexpression increases DJ-1 levels in melanoma cells. The direct interaction between α-Syn and DJ-1 may promote melanoma progression and represents a biomarker and therapeutic target for melanoma [123]. α-Syn expression has also been reported to inhibit ultraviolet B light-induced melanin synthesis in melanoma cells by suppressing melanogenesis [124, 125]. The mechanisms linking PD and melanoma also involve genetic mutations and oxidative stress [126]. PD-related genes, such as *PLA2G6* and *PARK2*, have been implicated in melanoma development [126, 127]. Immunohistochemical analysis of skin biopsies from patients with PD and melanoma showed α-Syn accumulation in the epidermis, particularly in melanocytes [128]. Epidemiological studies further suggest a bidirectional association, with increased risk of melanoma in PD patients and vice versa [129, 130].

Chronic inflammation plays a crucial role in neuronal dysfunction that underlies the onset of PD. There is increasing evidence linking PD with inflammatory skin conditions, including rosacea, bullous pemphigoid, and seborrheic dermatitis [131, 132], suggesting that skin inflammation and immune dysregulation may contribute to PD pathogenesis. Rosacea, a common inflammatory disorder in PD patients, is characterised by facial redness, burning sensations, pustules, telangiectasias, and flushing [133, 134]. To date, cohort and in vitro studies investigating the potential association between PD and rosacea remain limited [135–138]. However, a noteworthy study conducted in the Danish population with a 15-year follow-up, including healthy controls and rosacea patients but no PD cases, reported that patients with rosacea have a higher risk of developing PD, with onset occurring 2.4 years earlier than controls [137]. It has been hypothesized that the upregulation of matrix metalloproteinases (MMP-1, MMP-3 and MMP-9) represents a shared pathological mechanism between PD and rosacea [139].

Autoimmune skin diseases, such as bullous pemphigoid (BP), are linked to neurological diseases, including PD. BP is characterized by itching, redness, and papular lesions that can develop into vesicles and skin blisters [135, 140]. Interestingly, BP patients have a threefold increased risk of developing PD than the general population [141]. BP results from autoantibodies targeting hemidesmosomal proteins BPAG1 (Bullous pemphigoid antigen 1) and BPAG2 (Bullous pemphigoid antigen 2), which also have neuronal isoforms [141, 142]. The connection between PD and BP may lie in the production of antibodies directed against neuronal antigens, which by crossing a compromised BBB, cross-react with epidermal isoforms, potentially contributing to BP [143, 144]. Skin biopsies from BP patients show p-α-Syn positivity, regardless of PD diagnosis, suggesting a potential involvement of the ANS in both conditions. A gradual accumulation of p-α-Syn in skin nerve endings of BP patients without PD may represent a prodromal sign of neurodegenerative processes [145].

Seborrheic dermatitis (SD) is a common skin condition associated with PD [135, 146]. It is a chronic inflammatory disorder primarily affecting sebaceous gland-rich areas like the scalp and face [147]. While the exact cause of SD remains unclear, certain *Malassezia* species, common skin residents, are thought to play a key role [148] by metabolizing lipids into free fatty acids and hydrogen peroxide [135, 149, 150]. About one-third of PD patients experience SD symptoms long before PD diagnosis, with no effect on motor symptom onset [146]. SD may thus be an early sign of ANS involvement and a pre-motor PD marker [131, 151]. Changes in sebum production in PD may reflect systemic metabolic dysregulation. Blood lipidomic analyses have revealed significant shifts in the lipid profiles of PD patients, including alterations in fatty acids, phospholipids (such as ceramides), and sphingolipids like monosialodihexosylganglioside, which belongs to the ganglioside family [152–154]. Increased sebum production in PD patients alters skin odor and creates conditions favorable for the proliferation of *Malassezia* [155]. Additionally, elevated levels of *Malassezia globosa* and enzymes like phosphatase and lipase further increase the risk of developing SD in these patients [156]. Emerging evidence suggests that skin changes, such as alterations in sebum production and microbial flora, reflect shared pathological processes in PD and SD. PD patients exhibit increased seborrhea, altered sebum lipid profiles, and elevated volatile organic compounds (VOCs) [76, 157]. To date, no specific skin biomarkers directly linking PD and SD have been identified, and it remains unclear whether SD causes PD or vice versa. Nonetheless, lipidomic analyses of skin and blood provide valuable insights into the PD-related metabolic disturbances and may aid in early biomarker discovery.

## HD

HD is a genetic NDD caused by CAG repeat expansion in the *HTT* gene, which encodes the huntingtin (HTT) protein. The number of triplets correlates with disease severity and age of onset [158]. HD is characterised by chorea, psychiatric symptoms, and a decline of cognitive function [159, 160]. Neurodegeneration, particularly that in the striatum, results from intracellular accumulation of mutant huntingtin protein (mHTT), which forms toxic aggregates that impair cellular processes, including intracellular trafficking, vesicle transport, mitochondrial function, and synaptic activity [161–166].

mHTT has been detected in both CSF and blood, but is absent in non-HD patients lacking HTT gene mutations; therefore, it is a reliable disease-specific marker [167]. mHTT level in the CSF can be used to distinguish between healthy controls and HD mutation carriers [168]. However, signal overlap between mHTT, wildtype HTT and total HTT requires specific detection methods [169]. mHTT levels in the blood increase proportionally with disease progression [170]; however, detecting CNS-derived mHTT remains challenging. Since *HTT* is expressed throughout the body, the mutant protein is also present peripherally, though at lower levels than in the CNS. This widespread expression may interfere with the ability to distinguish between central and peripheral sources of mHTT [171]. Although HD primarily affects the brain, there has been a growing interest in recent years in peripheral tissues, including the skin, as alternative models for studying disease mechanisms and identifying biomarkers. One study using ELISA found elevated levels of t-tau in skin biopsies from HD patients, aligning with the diagnostic tests for cognitive, motor and genetic (CAG codon repeats) assessments. This suggests that detecting tau in the skin could support more accurate diagnosis [172].

HD development is also associated with systemic inflammation driven by pro-inflammatory cytokines and oxidative stress [173, 174]. Increased levels of IL-6, IL-8 and TNF-α indicative of CNS inflammatory response can also be observed in peripheral tissues [175]. Significant dysregulation of inflammatory-related genes and proteins has also been observed in skin, skeletal muscle and adipose tissue samples from HD patients [176].

Oxidative stress and mitochondrial dysfunction, well documented in the CNS, have also been detected in the peripheral tissues of patients with HD [177, 178]. Notably, mHTT associates directly with mitochondria, altering respiratory chain complexes II and IV, leading to reduced ATP production and increased oxidative stress [179]. mHTT also disrupts calcium homeostasis by promoting calcium release from the endoplasmic reticulum, thereby favouring mitochondria calcium uptake and facilitating the opening of the mitochondrial permeability transition pore, a potential trigger for apoptosis [180, 181].

Skin fibroblasts from HD patients exhibit increased production of mitochondrial superoxide (mtO₂⁻) along with elevated levels of antioxidant enzymes, including mitochondrial superoxide dismutase 2 (SOD2) and glutathione reductase (GR) [182]. Mitochondrial alterations linked to reduced oxidative phosphorylation and energy efficiency have also been observed in dermal fibroblasts from early-onset HD patients, regardless of sex or CAG repeat length [183]. Furthermore, these fibroblasts display a distinctive nuclear morphology associated with actin nuclear cap deficiency, which may serve as a cellular biomarker for HD diagnosis or disease monitoring [184]. Skin fibroblasts provide a convenient in vitro model for studying pathogenic mechanisms, as they can be differentiated into neurons showing neurodegenerative features [29]. However, no scientific studies have yet demonstrated a direct link between HD and skin diseases, highlighting the need for further research to corroborate the molecular evidence from fibroblast models.

## Advantages of using skin in NDD biomarker research

Biomarkers of NDDs can currently be obtained in fluids like CSF and blood. A wide range of target molecules can be extracted and analyzed from both sources. CSF, as the preferred biological matrix, allows us to observe processes occurring at the CNS level, including various pathological changes. However, lumbar punctures are very invasive and cannot be used routinely [185]. Blood sampling is cost-effective and rapid [186]; nevertheless, blood biomarker levels can be affected by comorbid conditions and external factors unrelated to neurodegenerative pathology, such as cardiovascular disease or metabolic disorders. The concentrations of biomarkers in the bloodstream vary greatly between individuals because several molecules are broken down by enzymes or bound to other molecules [22]. In addition, inflammatory cytokines may interfere with analysis, complicating accurate and reliable diagnosis [48]. Therefore, challenges remain in establishing standardized thresholds for normality [187]. Skin samples can be an alternative to blood as they are less affected by these biological constraints. Moreover, skin collection is performed through non-invasive or minimally invasive procedures, making it a more accessible and patient-friendly approach. As summarized in Table 2, skin represents a valuable and increasingly recognised source of biomarkers for NDDs and is emerging as a powerful diagnostic tool for protein aggregation diseases.

**Table 2:** Extra-brain biomarkers of neurodegenerative diseases, biological matrix, sources, and analytical approaches

NDDs	Biomarkers	Source/tissue	Detection method	References
Alzheimer’s disease	Aβ	Skin biopsy	Immunostaining on tissue sections and cell cultures	[27, 47–51]
	Aβ_42_, p-tau, t-tau	Human primary keratinocytes	Immunostaining	[27]
	Cortisol	Sweat	Hydrogel micropatches	[199]
Parkinson’s disease	α-Syn	Gastrointestinal tract, olfactory and salivary glands, pancreas, chest, scalp, abdomen, cervical skin, forearm, legs, melanocytes	Immunostaining on tissues and cell cultures	[11, 25, 87–89, 91, 101–107, 109, 110, 124, 128]
	α-Syn, o-α-Syn, p-α-Syn	Skin biopsy	Immuhistochemistry Immunofluorescence Western blotting	[25, 30, 102–108, 111, 145]
	Glutamate, hydrogen sulphide, LPS, SCFA, butyrate	Gut microbiota	RNA gene sequencing	[11, 91–93]
	*PLA2G6,* *PARK2, DJ-1*	Primary and metastatic cutaneous melanoma, melanoma cell lines, normal human epidermal melanocytes (NHEM)	Techniques of molecular analysis	[123, 126, 127]
	Lipases (C14, esterase lipase C8, esterase C4), phosphatases (naphthol-AS-BI-phosphohydrolase, acid phosphatase, alkaline phosphatase), fatty acids, cholesterol	Sebum	Sebutape Lipidomics	[76, 155, 156]
	VOCs (aldehydes, ketones and alcohols)	Skin/Sebum	Volatolomics	[157, 210–213]
Huntington’ disease	t-tau	Skin biopsies	ELISA assay Immunostaining	[27, 172]
	mtO_2_^−^, SOD2, GR	Skin fibroblasts	Mitochondrial metabolic assays, ROS detection, Western blotting	[182]
	Mitochondrial function and respiration parameters	Fibroblasts from skin biopsy	Metabolic assays	[183]
	Actin nuclear cap deficiency	Primary fibroblasts from skin biopsy	Morphological and molecular analysis	[184]

AD, Alzheimer’s disease; PD, Parkinson’s disease; HD, Huntington’s disease; Aβ, β‑amyloid; p‑tau/t‑tau, phosphorylated/total tau; α‑Syn, α‑synuclein; p‑α‑Syn, phosphorylated α‑synuclein; MMP-1/3/9, matrix metalloproteinase 1/3/9; VOCs, volatile organic compounds; LPS, lipopolysaccharide; SCFA, short‑chain fatty acids; mtO_2_^−^, mitochondrial superoxide; SOD2, superoxide dismutase 2; GR, glutathione reductase; PLA2G6, phospholipase A2 group VI

Compared to blood, skin biopsy offers a more direct and anatomically localized method for detecting the accumulation of pathological protein aggregates of α-Syn (p-α-Syn) [25], tau protein [188], and Aβ protein [19] within the dermal and epidermal compartments [27, 188–191]. In addition, skin biopsy enables histological analysis of peripheral nerve structure, providing valuable insights into the integrity of the PNS. For instance, the degeneration of intraepidermal nerve fibres may be an early event that reflects neurodegenerative processes in the CNS [192, 193]. Given the crucial role of biopsies in enabling early diagnosis of NDDs, considerable efforts are underway to develop standardized, highly sensitive assays for the detection of specific biomarkers [194]. However, despite promising advances, their full potential in routine clinical practice has yet to be definitively established.

It is also noteworthy that skin biopsies from patients with NDDs serve as a source for establishing primary cell cultures. These cultures are vital for in vitro studies focused on intracellular signalling pathways and molecular modulators involved in disease mechanisms. Studies on pathological primary fibroblasts [195, 196] have shown that these cells serve as an excellent model for replicating the aging process, a key factor in NDD development [197]. These cells can also be reprogrammed into induced pluripotent stem cells and differentiated into neurons [31], to mimic the pathological mechanisms of the nervous system.

**Fig. 1:**
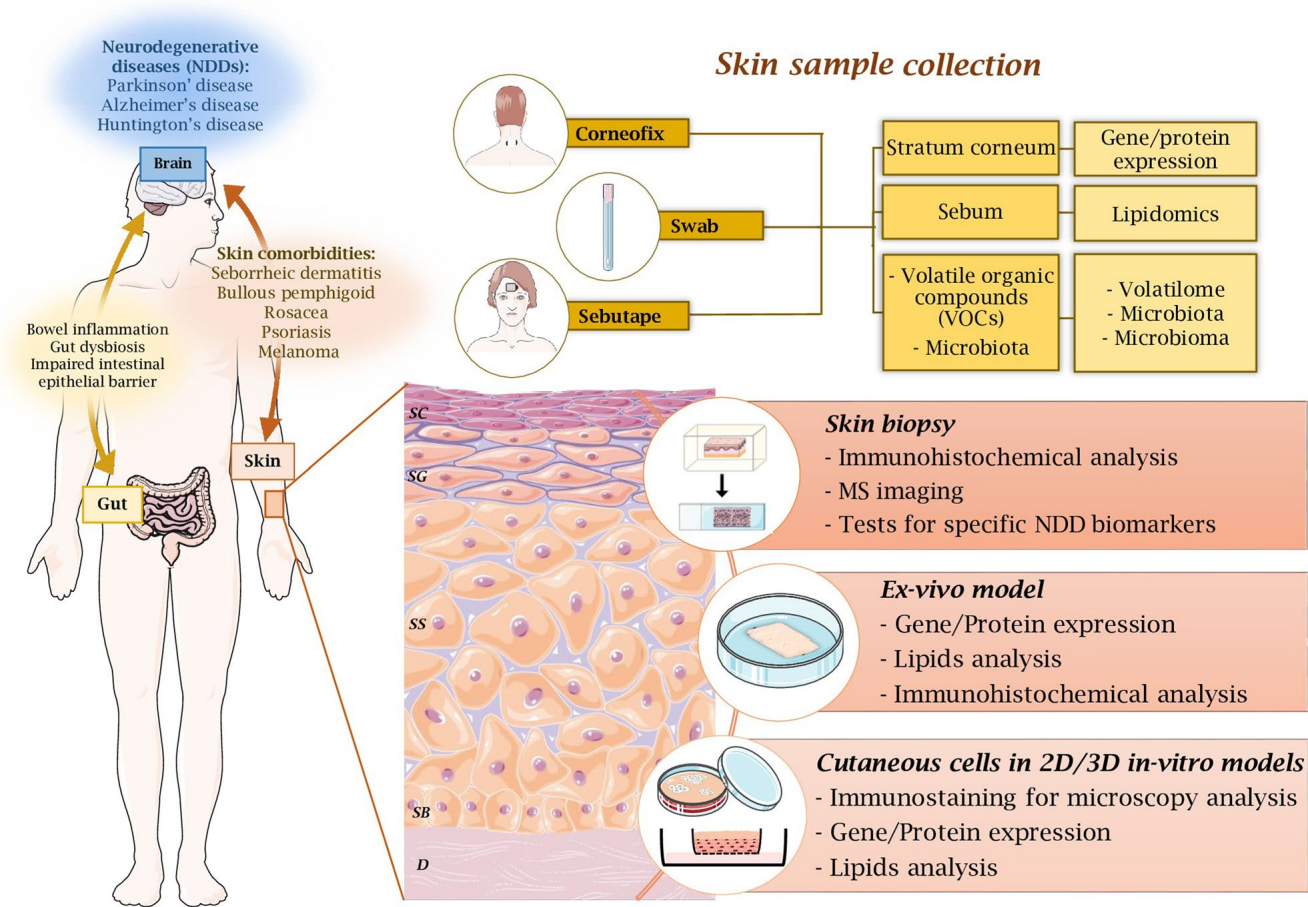
Strategies of using the skin as a mirror to investigate neurodegenerative processes and identify neurodegenerative disease biomarkers. Left, bidirectional connection between the brain and the skin, and a list of dermatological manifestations associated with NDDs, including seborrheic dermatitis, bullous pemphigoid, rosacea, psoriasis, and melanoma. Right, non-invasive techniques (corneofix, swab and sebutape) and minimally invasive techniques (skin biopsy) for collecting skin samples, including the stratum corneum, sebum and VOCs (volatile organic compounds), as well as setup of ex vivo and in vitro 2D/3D models. Various analytical approaches (e.g. gene/protein expression, lipidomics, volatilomics, microbiome analysis and microscopy) that are commonly used in dermatological translational research are also demonstrated

It is important to note that skin samples are especially valuable for advancing personalized medicine and enhancing the clinical management of NDDs and related dermatological conditions. Additionally, skin is a source of samples that can be easily collected and processed in various ways for different purposes, depending on the targets being studied. Figure 1 illustrates the association between NDDs and dermatological comorbidities, highlighting the techniques used for skin sample collection as well as the analytical methods employed to investigate pathological mechanisms and identify specific NDD biomarkers.

Tape stripping is the simplest method for collecting skin samples. This non-invasive, time-efficient procedure is widely used in dermatological research. It is especially valuable for studying stratum corneum biomarkers related to skin barrier function. Different amounts of proteins and mRNAs can be collected and analyzed using standard techniques such as Western blotting and real-time PCR by applying the adhesive tape and removing it after a set period [198]. This method directly reflects local pathology, as skin biomarkers offer information about ongoing local disease processes. Additionally, agarose-containing hydrogel micro-patches is an alternative non-invasive approach for mass spectrometry (MS) analysis, to study metabolites released from the skin [199].

Recently, the use of lipidomics for biomarker research has received growing interest, as changes in lipid composition and metabolism are linked to the development of NDDs [200]. Lipidomics is the systematic study of lipids, aiming to elucidate their functions and to assess both qualitative and quantitative alterations associated with disease states [201]. The review of Tong et al. provides a comprehensive summary of the role of lipid dysregulation in the pathogenesis of AD and PD, the importance of lipid monitoring and the development of new drug targets for lipid regulation [202].

Specifically, sebum lipidomics may be a useful tool for identifying NDD biomarkers. Sebutapes are adhesive patches that are applied to areas of interest on the skin, allowing different lipid classes, as well as inflammatory proteins and cytokines, to be easily analysed [203–209]. A recent study reported that sebum production and levels of certain lipids, such as palmitic acid, oleic acid, squalene, and cholesterol, are significantly higher in PD patients [76].

Volatilome, the entirety of VOCs produced by an organism, is another non-invasive source of skin biomarkers [210], as patients with PD exhibit a distinctive odour early in the disease. Analysis of the sebum of PD patients has revealed an altered composition of aldehydes, ketones and alcohols [157, 211–213]. Therefore, these minimally invasive techniques represent a new approach for detecting cutaneous NDD biomarkers.

## Conclusions

As the global population ages, NDDs are becoming increasingly prevalent, posing a significant public health burden. Early diagnosis remains elusive, and there is an urgent need for reliable biomarkers to detect NDDs in their initial stages, elucidate their molecular underpinnings, and guide therapeutic interventions at early stages.

The skin is a promising surrogate tissue for studying neurodevelopmental disorders, due to its shared embryological origin with the brain, molecular signaling, and accessibility. Its role as a "mirror" of dysfunction occurring in the CNS is emphasized by growing evidence linking NDDs to various dermatological conditions, including melanoma and inflammatory skin disorders. The shared molecular mechanisms, such as oxidative stress, mitochondrial and lysosomal dysfunction, abnormal lipid metabolism, and chronic inflammation, further strengthen this connection.

Studies of skin biopsies and cell models using immunohistochemistry and multi-omics technologies provide a unique opportunity to detect early pathogenic changes and identify clinically relevant biomarkers. Notably, skin sampling offers a minimally invasive way for long-term monitoring, which could transform how we detect and treat NDDs. To fully realize this potential, future research must focus on standardizing methods and ensuring data reproducibility. Incorporating skin-based diagnostics into clinical practice could lead to a major shift in NDDs care, enabling earlier intervention, personalized treatments, and improved outcomes.

## Data Availability

Not applicable.

## Abbreviations

NDDs: Neurodegenerative diseases
HD: Huntington’s disease
AD: Alzheimer’s disease
PD: Parkinson’s disease
ALS: Amyotrophic lateral sclerosis
CNS: Central nervous system
PNS: Peripheral nervous system
ANS: Autonomic nervous system
BBB: Blood–brain barrier
CSF: Cerebrospinal fluid
Aβ: β-Amyloid
t-tau: Total tau
p-tau: Phosphorylated tau
α-Syn: α-Synuclein
p-α-Syn: Phosphorylated α-synuclein
o-α-Syn: Oligomeric α-synuclein
PSEN: Presenilin
APP: Amyloid precursor protein
TNF-α: Tumor necrosis factor-alpha
IL: Interleukin
ROS: Reactive oxygen species
APOE Ɛ4: Apolipoprotein Ɛ4
HPA: Hypothalamic-pituitary-adrenal axis
SNCA: Synuclein alpha
LRRK2: Leucine-rich repeat kinase 2
PINK1: PTEN-induced kinase 1
PARK2: Parkin 2
DJ-1: Protein deglycase DJ-1
GBA: Glucocerebrosidase
APs: Atypical parkinsonisms
MSA: Multiple system atrophy
CBD: Corticobasal degeneration
PSP: Progressive supranuclear palsy
BP: Bullous pemphigoid
BPAG: Bullous pemphigoid antigen
SD: Seborrheic dermatitis
VOCs: Volatile organic compounds
mHTT: Mutant huntingtin
MS: Mass spectrometry
